# Functional neuroanatomy of speech signal decoding in primary progressive aphasias

**DOI:** 10.1016/j.neurobiolaging.2017.04.026

**Published:** 2017-08

**Authors:** Chris J.D. Hardy, Jennifer L. Agustus, Charles R. Marshall, Camilla N. Clark, Lucy L. Russell, Emilie V. Brotherhood, Rebecca L. Bond, Cassidy M. Fiford, Sasha Ondobaka, David L. Thomas, Sebastian J. Crutch, Jonathan D. Rohrer, Jason D. Warren

**Affiliations:** a Dementia Research Centre, Department of Neurodegenerative Disease, UCL Institute of Neurology, University College London, London, UK; b Wellcome Centre for Neuroimaging, UCL Institute of Neurology, University College London, London, UK; c Leonard Wolfson Experimental Neurology Centre, UCL Institute of Neurology, University College London, London, UK

**Keywords:** Frontotemporal dementia, Primary progressive aphasia, Semantic dementia, Logopenic aphasia, Progressive nonfluent aphasia, Functional magnetic resonance imaging

## Abstract

The pathophysiology of primary progressive aphasias remains poorly understood. Here, we addressed this issue using activation fMRI in a cohort of 27 patients with primary progressive aphasia (nonfluent, semantic, and logopenic variants) versus 15 healthy controls. Participants listened passively to sequences of spoken syllables in which we manipulated 3-key auditory speech signal characteristics: temporal regularity, phonemic spectral structure, and pitch sequence entropy. Relative to healthy controls, nonfluent variant patients showed reduced activation of medial Heschl's gyrus in response to any auditory stimulation and reduced activation of anterior cingulate to temporal irregularity. Semantic variant patients had relatively reduced activation of caudate and anterior cingulate in response to increased entropy. Logopenic variant patients showed reduced activation of posterior superior temporal cortex to phonemic spectral structure. Taken together, our findings suggest that impaired processing of core speech signal attributes may drive particular progressive aphasia syndromes and could index a generic physiological mechanism of reduced computational efficiency relevant to all these syndromes, with implications for development of new biomarkers and therapeutic interventions.

## 1 Introduction

The primary progressive aphasias (PPAs) have collectively helped establish the paradigm of selective neural vulnerability to neurodegenerative pathologies (Mesulam, 1982, Mesulam et al., 2014). These disorders have been characterized as ‘language-led dementias’, comprising 3 canonical syndromes (Gorno-Tempini et al., 2011): nonfluent variant PPA (nfvPPA), presenting with impaired speech production and/or agrammatism; semantic variant PPA (svPPA), presenting with impaired single-word comprehension and vocabulary loss due to progressive erosion of semantic memory; and logopenic variant PPA (lvPPA), presenting with word-finding difficulty and impaired auditory verbal working memory. These syndromes have separable though partly overlapping neuroanatomical and pathological substrates: nfvPPA principally targets a peri-Sylvian brain network and svPPA an anterior temporal lobe network and both syndromes are generally underpinned by non-Alzheimer proteinopathies in the frontotemporal lobar degeneration spectrum (Grossman, 2012, Hodges and Patterson, 2007, Rohrer et al., 2011); whereas lvPPA targets a network centered on the temporo-parietal junction and is most often underpinned by Alzheimer pathology (Gorno-Tempini et al., 2008, Rabinovici et al., 2008, Rohrer et al., 2010).

The pathophysiological basis of PPA remains to be fully defined (Grossman, 2012, Mesulam et al., 2014). Language impairment is the dominant clinical consideration in PPA and enshrined in current consensus diagnostic criteria (Gorno-Tempini et al., 2011). However, a substantial proportion of cases of PPA do not fall clearly into current diagnostic categories, whereas similar linguistic deficits may be prominent in other dementia syndromes such as bvFTD (Hardy et al., 2015, Rohrer and Warren, 2016). A number of studies have documented profiles of nonverbal auditory deficits associated with PPA syndromes (Bozeat et al., 2000, Fletcher et al, 2015, Golden et al., 2015, Golden et al., 2016, Goll et al., 2010, Goll et al., 2011, Grube et al., 2016, Hailstone et al., 2011, Hailstone et al., 2012, Hardy et al., 2016, Rohrer et al., 2012). Indeed, presentations with word deafness and auditory agnosia have been well-attested since the earliest descriptions of PPA (Fletcher et al., 2013, Mesulam, 1982, Serieux, 1893, Uttner et al., 2006). This is likely to reflect shared neural resources for processing speech and other complex auditory signals, consistent with evidence in the healthy brain (Binder et al., 2000, Goll et al., 2012, Griffiths and Warren, 2002, Hardy et al., 2016, Warren and Griffiths, 2003). It has been proposed that generic deficits of auditory signal processing may be intrinsic to PPA syndromes and could underpin neurolinguistic impairment in these syndromes (Goll et al., 2010, Grube et al., 2016).

Functional MRI (fMRI) has delineated altered (including compensatory) patterns of cerebral activation in PPA cohorts relative to healthy controls (Goll et al., 2012; Nelissen et al., 2011, Vandenbulcke et al., 2005, Wilson et al., 2010, Wilson et al., 2016). However, this technique has not been used previously to identify fundamental mechanisms of abnormal information processing in PPA. Here, we used activation fMRI to deconstruct the functional neuroanatomy of speech perception in PPA into component neural mechanisms that process core attributes of speech signals. We studied a cohort of patients representing all major PPA syndromes in relation to healthy older individuals. In experimental stimuli based on sequences of spoken syllables, we manipulated 3 generic auditory speech signal characteristics relevant to previously documented neurolinguistic deficits in PPA syndromes: temporal regularity, phonemic structure (presence of intelligible phonemes), and average signal information content (entropy).

Analysis of temporal structure is crucial for speech segmentation (and therefore lexical access) in healthy individuals (Dilley and McAuley, 2008, Dilley et al., 2010) and vulnerable particularly in nfvPPA (Grube et al., 2016). In this experiment, we varied syllabic timing such that the interval between syllables was either regular (isochronous) or irregular (anisochronous). Phonemes are the smallest intelligible units of spoken language and constitute a special category of auditory ‘objects’ (Griffiths and Warren, 2004), defined by specific combinations of spectrotemporal acoustic features: phonemic processing deficits are prominent in lvPPA and nfvPPA (Hailstone et al., 2012, Hardy et al., 2015, Henry et al., 2016, Rohrer et al., 2010). Here, we manipulated higher-order spectral structure that distinguishes natural (intelligible) phonemes from complex synthetic (unintelligible) speech-like sounds (Blesser, 1972), to target a universal neural mechanism of phoneme detection relevant to any language. ‘Entropy’ is a concept derived from information theory describing the average amount of information carried by any signal (Overath et al., 2007): it measures signal unpredictability, in the sense that an unpredictable signal is less ‘redundant’ and therefore conveys more information (henceforth in this article, we use information in this technical sense). We manipulated the information content (entropy) of our experimental stimuli by varying the predictability of pitch patterns across successive syllables in a sequence, a generic characteristic related to speech prosody but not bound to the prosodic conventions of any particular language. Deficits of pitch pattern processing have been documented in all major PPA syndromes (Golden et al., 2015, Golden et al., 2016, Hsieh et al., 2011, Rohrer et al., 2012): however, the experimental manipulation used here (unlike those previously employed) was designed to index a brain mechanism responsible for computing the overall statistics of an auditory object (the ‘melody’ of the syllable sequence). An analogous computational mechanism has been invoked to account for the profile of evolving object recognition deficits across sensory modalities in svPPA (Lambon Ralph et al., 2010).

To assess the effect of PPA syndromes on these generic mechanisms of speech signal analysis relatively uncontaminated by executive, working memory or other extraneous task demands (Hickok and Poeppel, 2007, Rauschecker and Scott, 2009), we adopted a passive listening fMRI paradigm with ‘sparse’ image acquisition (presentation of auditory stimuli interleaved with scanner noise). We hypothesized that PPA syndromes would have separable functional neuroanatomical signatures of abnormal speech signal decoding relative to healthy older individuals. Based on available evidence in PPA and in the healthy brain, we further hypothesized that nfvPPA and lvPPA would show abnormal processing of speech signal isochrony and phonemic structure (Grube et al., 2016, Hailstone et al., 2012, Hardy et al., 2015, Henry et al., 2016, Rohrer et al., 2010), whereas svPPA would show abnormal processing of entropy as an auditory object statistic (Golden et al., 2015, Hsieh et al., 2011, Lambon Ralph et al., 2010). Finally, we hypothesized that the functional substrates of isochrony and entropy processing would lie within a distributed network including posterior temporal, cingulate and striatal structures, previously implicated in the analysis of auditory regularity and predictability (Cope et al., 2014, Griffiths and Warren, 2002, Ide et al., 2013, Overath et al., 2007); whereas the substrate of phoneme processing would lie within superior temporal cortex, previously implicated in the analysis of phonemic structure (Hickok and Poeppel, 2007, Liberman and Mattingly, 1989, Rauschecker and Scott, 2009, Scott et al., 2000).

## 2 Materials and methods

### 2.1 Participants

The patient cohort comprised 12 patients with nfvPPA (5 female; mean age 70.9 years), 9 patients with svPPA (3 female; mean age 62.3 years), and 6 patients with lvPPA (2 female; mean age 62.7 years), each fulfilling consensus criteria for the respective syndromic diagnosis (Gorno-Tempini et al., 2011) and recruited via a specialist cognitive disorders clinic. Brain magnetic resonance imaging (MRI) findings corroborated the syndromic diagnosis in each case; no patient had radiological evidence of significant comorbid cerebrovascular damage. Cerebrospinal fluid tau/abeta profiles were available for 5 of the 6 patients with lvPPA, all of which were consistent with Alzheimer's pathology based on local reference ranges (total tau: beta-amyloid_1–42_ ratio >1). Fifteen healthy older individuals (8 female; mean age 68.8 ± 4.5 years) with no history of neurological or psychiatric illness also participated. All participants had a comprehensive general neuropsychological assessment. Demographic, clinical, and neuropsychological characteristics of participant groups are summarized in Table 1. Peripheral hearing function was assessed in all participants using pure tone audiometry (procedural details in Supplementary Material on-line).

**Table 1:** Demographic, clinical, and neuropsychological characteristics of participant groups

Characteristic	Controls	nfvPPA	svPPA	lvPPA
Demographic and clinical				
No. (m:f)	7:8	7:5	6:3	4:2
Age (yrs)	68.8 (4.5)	70.9 (8.6)	**62.3 (5.7)**^h^	**62.7 (5.8)**^h^
Handedness (R:L)	14:1	10:2	8:1	5:1
Education (y)	16.4 (2.6)	14.8 (2.9)	14.9 (2.9)	14.3 (3.1)
MMSE (/30)	29.8 (0.4)	**24.4 (5.2)**	**19.8 (9.3)**	**16.0 (8.8)**^h^
Symptom duration (y)	-	4.9 (2.6)	5.0 (2.7)	4.7 (1.6)
PTA best ear (N:Mild:Mod)	8:7:0	3:6:2^a^	5:3:0^a^	3:3:0
General intellect: IQ				
WASI verbal IQ	126.7 (7.3)	**84.5 (23.6)**^a^	**70.9 (7.3)**	**68.8 (20.9)**
WASI performance IQ	126.1 (9.8)	**97.0 (22.2)**	**101.4 (25.2)**	**86.0 (15.4)**
Episodic memory				
RMT words (/50)	49.5 (0.9)	**42.5 (6.8)**^a^	**35.3 (8.5)**^b^	**34.0 (11.9)**^b^
RMT faces (/50)	45.5 (2.9)	**38.8 (5.8)**	**32.0 (5.9)**^b,^^h^	**34.8 (7.4)**
Camden PAL (/24)	20.7 (3.3)	**15.0 (8.4)**^b^	**3.4 (4.0)**^b,^^h^	**3.6 (6.1)**^a,^^h^
Working memory				
WMS-R digit span forward (max)	7.3 (1.0)	**4.9 (1.1)**^c^	6.2 (2.0)	**3.0 (0.6)**^h^^,^^i^
WMS-III spatial span forward (max)	5.5 (1.0)^b^	**4.3 (1.0)**^d^	5.4 (0.9)	**3.5 (0.8)**^h^^,^^i^
Executive skills				
WASI block design (/71)	45.8 (12.4)	**21.3 (18.5)**	33.6 (23.3)	**15.7 (16.4)**
WASI matrices (/32)	27.3 (2.3)	**15.9 (8.7)**	**19.3 (10.5)**	**14.0 (6.7)**
WMS-R digit span reverse (max)	5.7 (1.2)	**3.0 (1.4)**^c,^^i^	4.4 (2.1)	**1.8 (1.5)**^i^
WMS-III spatial span reverse (max)	5.6 (0.9)^b^	**4.1 (1.6)**^d^	4.7 (1.9)	**3.0 (1.3)**^i^
Letter fluency (F: total)	17.4 (4.6)	**5.5 (5.8)**^f^	**7.3 (6.3)**^a^	**2.2 (1.8)**^a^
Category fluency (animals: total)	25.3 (5.1)	**10.7 (4.3)**^e^	**5.2 (5.7)**	**5.0 (3.5)**^a,^^h^
Trails A (s)	34.2 (5.3)	**90.7 (49.4)**^b,^^i^	46.9 (19.3)^a^	**126.2 (96.2)**^i^
Trails B (s)	73.5 (18.0)	**221.2 (90.9)**^b,^^i^	126.9 (86.0)^a^	**221.0 (92.2)**
Posterior cortical skills				
GDA calculation (/24)	14.7 (5.9)	**5.0 (3.9)**^d^	9.8 (8.8)	**1.7 (5.9)**^i^
VOSP object decision (/20)	18.9 (1.4)	**15.2 (4.1)**^a^	**16.3 (3.2)**^a^	**16.7 (2.3)**
Neurolinguistic skills				
Auditory input processing				
PALPA-3 (/36)	35.8 (0.6)^b^	**33.3 (3.2)**^d^	**32.0 (6.5)**	**31.2 (3.9)**
Word retrieval				
GNT (/30)	26.3 (2.7)	**15.6 (7.8)**^a^	**1.9 (4.6)**^h^	**4.7 (7.2)**^h^
BNT (/30)	29.7 (0.7)^c^	**20.6 (8.9)**^e^	**5.3 (7.1)**^h^	**9.3 (7.7)**^h^
Speech comprehension				
BPVS (/51)	49.5 (1.4)	**42.1 (8.0)**	**9.6 (15.8)**^a,^^g^^,^^h^	**34.2 (14.7)**
Concrete synonyms (/25)	24.3 (0.9)^b^	21.1 (4.7)^c^	**14.2 (3.2)**^g^^,^^h^	**17.8 (3.1)**^a^
Abstract synonyms (/25)	24.4 (1.0)^b^	**20.8 (5.0)**^c^	**15.5 (3.5)**^a,^^h^	**15.8 (4.5)**^a^
PALPA-55 sentences (/24)	23.7 (0.6)^d^	**21.1 (4.2)**^d^	**19.4 (6.7)**	**13.7 (5.1)**^h^
Speech repetition				
Polysyllabic words (/45)	44.5 (0.9)^b^	**27.7 (17.3)**^f,^^i^	43.8 (1.6)	**32.2 (7.0)**^i^
Short sentences (/10)	10.0 (0.0)^c^	**5.0 (4.7)**^f,^^i^	**9.6 (0.7)**^a^	**3.5 (3.1)**^a,^^i^
Spelling				
GST (/30)	26.8 (1.7)^b^	**16.1 (9.3)**^e^	**11.5 (9.8)**^a^	**8.6 (5.7)**^a^
Post-scan behavioral tasks^j^				
Temporal regularity (/20)	19.7 (0.8)	**17.3 (3.2)**^a^	18.4 (2.8)^a^	**16.7 (4.5)**
Phonemic structure (/20)	19.0 (1.4)	**14.8 (3.6)**^a^	**15.1 (3.9)**^a^	**12.3 (1.3)**
Entropy (/20)	19.3 (1.1)	**13.6 (3.2)**^a^	**14.9 (4.3)**^a^	**13.2 (3.4)**

Mean (standard deviation) values are shown. Raw scores are presented, with the maximum value possible in parentheses, unless otherwise indicated. Significant differences (*p* < 0.05) from healthy control values are in bold. Reduced numbers of participants are indicated: ^a^n − 1; ^b^n − 2; ^c^n − 3; ^d^n − 4; ^e^n − 5; ^f^n − 6.

Key: BNT, Boston Naming Test; BPVS, British Picture Vocabulary Scale; Controls, healthy control group; D-KEFS, Delis-Kaplan Executive Function System; GDA, Graded Difficulty Arithmetic test; GNT, Graded Naming Test; GST, Graded Spelling Test; lvPPA, patient group with logopenic variant primary progressive aphasia; Mild, mild hearing loss; MMSE, Mini–Mental State Examination; Mod, moderate hearing loss; N, normal hearing; NART, National Adult Reading Test; nfvPPA, patient group with nonfluent variant primary progressive aphasia; PAL, paired associates learning; PALPA, Psycholinguistic Assessments of Language Processing in Aphasia; PTA, pure tone audiometry; RMT, Recognition Memory Test; svPPA, patient group with semantic variant primary progressive aphasia; VOSP, Visual Object and Space Perception Battery (Object Decision); WAIS, Wechsler Adult Intelligence Scale; WASI, Wechsler Abbreviated Scale of Intelligence; WMS, Wechsler Memory Scale.

g Significantly different from lvPPA group.

h Significantly different (*p* < 0.05) from nfvPPA group.

i Significantly different from svPPA group.

j See text for details.

All participants gave informed consent, and the ethical approval for the study was granted by the National Hospital for Neurology and Neurosurgery and University College London Research Ethics Committees, following Declaration of Helsinki guidelines.

### 2.2 Experimental stimuli

**Fig. 1:**
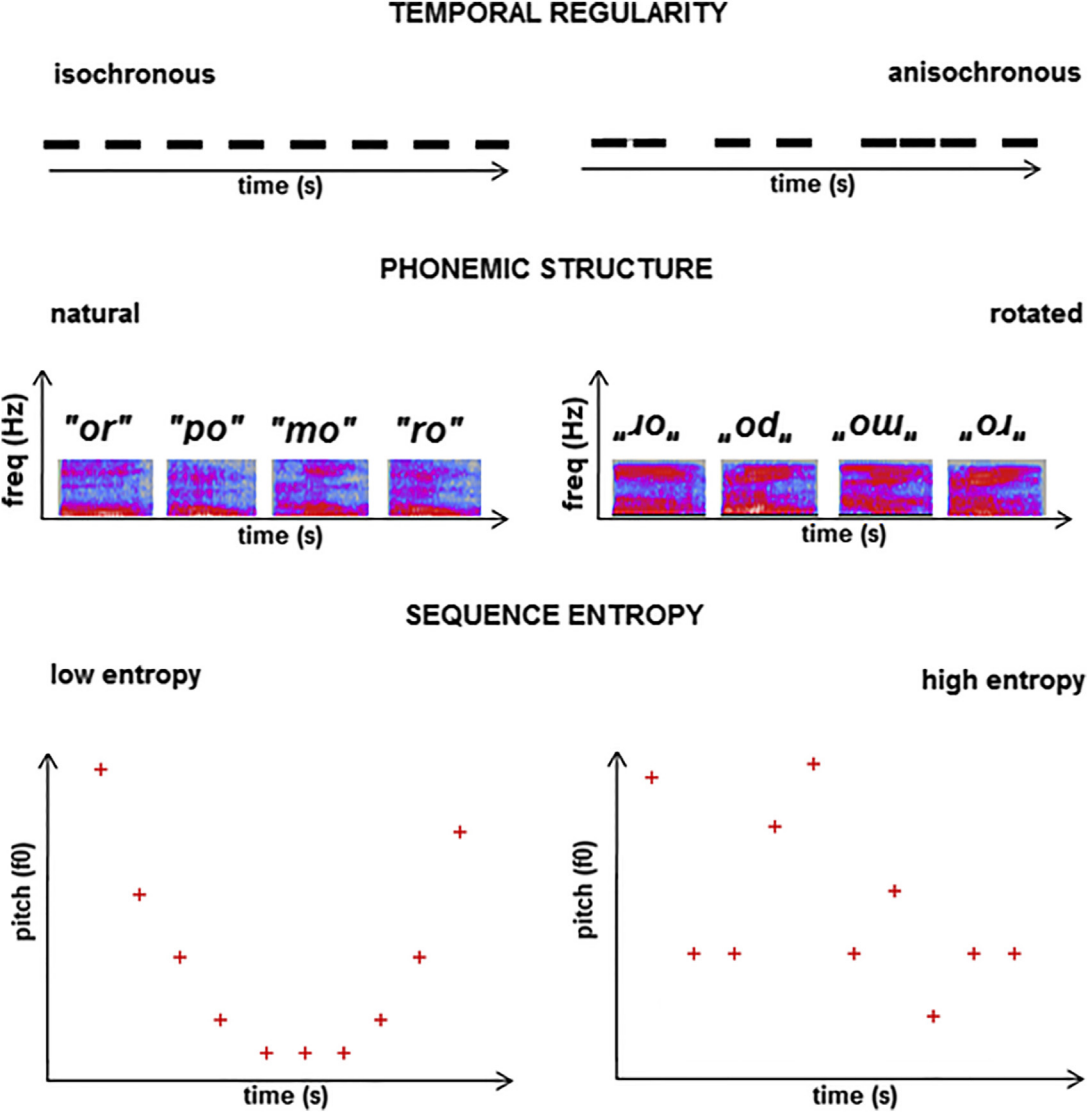
Schematic representations of stimulus manipulations used to create the conditions in the fMRI experiment (see text for details). The top panels show examples of isochronous and anisochronous sequences. The middle panels show spectrograms for syllable sequences in the natural and spectrally rotated conditions. The bottom panels show examples of low and high entropy sequences, based on degree of correlation between pitch (fundamental frequency, f0) of successive intervals (highly correlated and approaching a sine wave contour in the low entropy condition; uncorrelated in the high entropy condition). Using these manipulations, 8 types of experimental trials were created: (1) isochronous—natural speech—high entropy; (2) isochronous—natural speech—low entropy; (3) isochronous—rotated speech—high entropy; (4) isochronous—rotated speech—low entropy; (5) anisochronous—natural speech—high entropy; (6) anisochronous—natural speech—low entropy; (7) anisochronous—rotated speech—high entropy; and (8) anisochronous—rotated speech—low entropy. Combining these trial types allowed contrasts between the conditions representing a particular experimental manipulation while balancing for each of the other manipulations (see text).

The stimuli presented in the fMRI experiment were based on sequences of spoken syllables comprising consonant-vowel or vowel-consonant phoneme combinations, recorded in a standard Southern English accent by a young adult male speaker. The syllables ‘af’, ‘ba’, ‘da’, ‘mo’, ‘om’, ‘or’, ‘po’, and ‘ro’ were selected for high intelligibility and identifiability, based on pilot testing in 5 young adult listeners in our laboratory. In MATLAB R2012a (https://uk.mathworks.com/), recorded syllables were each edited to duration 240 msec and concatenated with random ordering into sequences; each sequence comprised 20 syllables and intervening silent intervals, with fixed overall sequence duration (7.65 seconds) and root-mean-square intensity. We varied 3 sequence parameters independently to create the experimental conditions. Temporal regularity was manipulated by varying the inter-syllable interval within each sequence such that the interval was either kept constant at 150 ms (isochronous condition) or randomly allocated in the range 50–250 msec around a mean of 150 msec (anisochronous condition), maintaining the same overall sequence tempo. Phonemic structure was manipulated by spectrally rotating spoken syllables using a previously described procedure (Blesser, 1972); spectral rotation preserves overall acoustic spectral and temporal complexity and bandwidth but radically alters spectral detail, by inverting the acoustic frequency spectrum. This manipulation renders the rotated signal unintelligible as human speech (it is perceived as ‘alien’ or ‘computer speech’) and here enabled us to create stimulus conditions in which the constituent syllables in each sequence were either all unrotated (natural) or all spectrally rotated (unintelligible). Entropy or average information content in the sequence was manipulated by varying fundamental frequency (pitch) of constituent syllables over a half-octave range from a lower fundamental frequency of 100 Hz with a 20-note octave division (i.e., not conforming to the intervals of Western music), adapting a previously described procedure (Overath et al., 2007). Pitch sequences were based on inverse Fourier transforms of f^n^ power spectra, using values of n = 0 (no correlation between successive pitch values) for the high entropy condition and n = 4 (high correlation between successive pitch values, approaching a sine wave contour) for the low entropy condition. The stimulus manipulations are schematized in Fig. 1; examples of stimuli are in Supplementary Material online.

### 2.3 Functional MRI protocol

#### 2.3.1 Stimulus delivery

During fMRI scanning, stimuli were presented in randomized order via a notebook computer running the Cogent v1.32 extension of MATLAB (www.vislab.ucl.ac.uk/cogent_2000.php). Each stimulus trial was triggered by the MR scanner on completion of the previous MR image acquisition in a sparse acquisition protocol. Stimuli were played binaurally via electrodynamic headphones (www.mr-confon.de) at a comfortable listening level (at least 70 dB). Twenty stimulus trials were administered for each of 8 trial types (Fig. 1): across trial types, the contrasts of interest were constructed by comparing conditions that differed in the speech signal parameter of interest (temporal regularity, 80 isochronous vs 80 anisochronous trials; phonemic structure, 80 natural vs 80 spectrally rotated trials; information content, 40 high vs 40 low entropy trials, assessed separately for natural and spectrally rotated speech stimuli). In addition, there were 20 silent ‘rest’ trials, yielding a total of 180 trials for the experiment for each participant. Participants were instructed to lie quietly and listen to the sounds with eyes lightly closed; there was no in-scanner output task.

#### 2.3.2 Brain image acquisition

Functional MRI scans were acquired using a 12-channel RF receive head coil on a 3T Siemens Tim Trio MRI scanner. The EPI sequence comprised 48 oblique transverse slices covering the whole brain (slice thickness 2 mm, interslice gap 1 mm, 3 mm in-plane resolution, slice TR/TE 70/30 ms, echo spacing 0.5 ms, matrix sixe 64 × 64 pixels, FoV 192 × 192 mm, phase encoding direction anterior-posterior) with slice tilt −30° (T>C). Sparse-sampling EPI acquisition with repetition time 11.36 seconds (corresponding to an inter-scan interval of 8 seconds) was used to reduce any interaction between scanner acoustic noise and auditory stimulus presentations. One initial brain volume was acquired to allow equilibration of longitudinal T1 magnetization and discarded from further analysis. A B0 field-map was also acquired (TR = 688 ms; TE1 = 4.92 ms, TE2 = 7.38 ms, 3 × 3 × 3 mm resolution, no interslice gap; matrix size = 80 × 80 pixels; FoV = 240 × 240 mm; phase encoding direction = A-P) to allow post-processing geometric correction of EPI data for B0 field inhomogeneity distortions.

To enable structural coregistration and comparison with activation data, volumetric brain MRI scans were acquired for each participant on the same scanner using a 32-channel phased array head coil and a T_1_-weighted sagittal 3D magnetization rapid gradient echo sequence (TE = 2.9 ms, TR = 900 ms, TI = 2200 ms), with a 256 × 256 × 208 acquisition matrix and voxel size 1.1 × 1.1 × 1.1 mm.

#### 2.3.3 Post-scan behavioral testing

After the scanning session, each participant's ability to perceive the key experimental manipulations was determined using psychoacoustic tests employing 2-alternative-forced-choice decisions on the syllable sequences presented during scanning. Separate tests were administered to assess temporal processing (regular vs. irregular sequences), phoneme detection (natural vs. artificial [spectrally rotated] phonemes) and pitch pattern detection (low entropy vs. high entropy sequences). Pictorial cards were used to ensure all participants understood the task instructions and to allow nonverbal responses where preferred (details of task instructions and aids used are in Fig. S1 in Supplementary Material on-line). For each test, 20 stimuli (10 representing each of the 2 conditions of interest) were presented; no feedback was given and no time limits were imposed. Participants' responses were recorded for offline analysis.

### 2.4 Data analyses

#### 2.4.1 Analysis of clinical and behavioral data

All analyses were performed in Stata version 14.1. Demographic and other clinical variables were compared between groups using 2-sample *t*-tests for continuous variables and Fisher's exact tests for categorical variables. Given the nonnormative distribution of residuals for neuropsychological data, nonparametric Mann-Whitney U tests were used to compare groups for performance on behavioral tests. Peripheral hearing was analyzed by creating a composite pure tone average score comprising the average of the levels (dB) required for tone detection at 500, 1000, and 2000 Hz for each ear separately. Using data from each participant's “best” ear, scores within the range 0–25 dB were categorized as “normal”, 26–40 dB as “mild hearing loss”, and 41–55 dB as “moderate hearing loss”. Using these classifications for each participant as a categorical variable, Fisher's exact test was again used to compare groups. A threshold of *p* < 0.05 was accepted as the criterion of statistical significance for all reported tests.

#### 2.4.2 Analysis of fMRI data

Functional MRI data were analyzed using statistical parametric mapping software (SPM12; www.fil.ion.ucl.ac.uk/spm). During initial image preprocessing, the EPI functional series for each participant was realigned to the first image. Images were unwarped incorporating field-map distortion information (Hutton et al., 2002). All individual functional images were spatially registered to a group mean template image using the DARTEL toolbox (Ashburner, 2007) and then normalized to Montreal Neurological Institute (MNI) standard stereotactic space. To construct the group brain template, each individual T1-weighted MR brain image was first coregistered to the corresponding EPI series and segmented into gray matter, white matter, and cerebrospinal fluid. Functional images were smoothed using a 6 mm full-width-at-half-maximum Gaussian kernel, with voxel volume 3 × 3 × 3 mm. For visualization of results, a study-specific mean structural brain image template was created by warping all bias-corrected native space whole-brain images to the final DARTEL template and calculating the average of the warped images. An explicit mask was created using an automatic-mask creation strategy so that only appropriate voxels would be included in the resultant analyses (Ridgway et al., 2009).

Preprocessed functional images were entered into a first-level design matrix incorporating the experimental conditions modeled as separate regressors convolved with the standard hemodynamic response function and also including 6 head movement regressors generated from the realignment process. For each participant, first-level *t*-test contrast images were generated for the main effects of auditory stimulation (any sound vs. silence); temporal regularity (isochronous > anisochronous sequences); phonemic structure (natural speech > spectrally rotated speech); and fundamental signal information content (high entropy > low entropy sequences), separately for natural and spectrally rotated speech conditions (since the decoding of pitch pattern is likely a priori to differ for speech signals with dissimilar spectral structure). Both “forward” and “reverse” contrasts were assessed in each case. Contrast images for each participant were entered into a second-level full factorial model in which effects within each participant group and differences between patient and healthy control groups were explored using *t*-test contrasts.

Contrasts were assessed at a cluster-level significance threshold of *p* < 0.05 after family-wise error (FWE) correction for multiple comparisons over the whole brain and at a peak-level significance threshold of *p* < 0.05_FWE_ within 2 prespecified neuroanatomical regions of interest in each cerebral hemisphere, in line with neuroanatomical evidence from previous studies. Correlates of speech temporal regularity and sequence information content (entropy) processing were assessed within a region comprising posterior superior temporal gyrus and sulcus, planum temporale, dorsal striatum, and anterior cingulate cortex (Cope et al., 2014, Griffiths and Warren, 2002, Ide et al., 2013, Overath et al., 2007); whereas correlates of phonemic processing were assessed within a more restricted subregion comprising planum temporale and posterior to mid superior temporal gyrus and sulcus (Hickok and Poeppel, 2007, Liberman and Mattingly, 1989, Rauschecker and Scott, 2009, Scott et al., 2000). Anatomical regions were derived from Oxford-Harvard cortical maps via FSLView (Desikan et al., 2006, Jenkinson et al., 2012) and edited in MRICro (http://www.mccauslandcenter.sc.edu/crnl/mricro) to conform to the study-specific template brain image. Regions of interest are presented in Fig. S2 in Supplementary Material on-line.

For experimental contrasts of interest in analyses directly comparing the healthy control group with each patient group, linear regression models were used to assess any correlation of effect size (beta parameter) with performance on the corresponding post-scan behavioral task across the 2 groups.

#### 2.4.3 Analysis of structural MRI data

To compare functional with structural brain changes in our PPA cohort, the distribution of regional disease-related gray-matter atrophy was assessed in each patient group using voxel-based morphometry (VBM). Both for patients and heathy controls, structural MRI segmentation, modulation, and normalization to MNI space were performed using default parameters in SPM12 in conjunction with the DARTEL toolbox (Ashburner, 2007), with a Gaussian smoothing kernel of 6-mm full-width-at-half-maximum. Group mean template images and group-specific explicit masks were created using the procedures outlined above. Each patient group was then compared with the healthy control group using 2-sample *t*-tests including covariates of age, gender, and total intracranial volume at a lenient voxel-wise threshold *p* < 0.001 uncorrected over the whole brain. Local maxima of gray-matter atrophy and functional change relative to the healthy control reference were systematically compared for each syndromic group, to assess whether these fell within the same functional brain regions.

## 3 Results

### 3.1 General participant characteristics

Participant groups did not differ in gender, handedness, or educational attainment (all *p* > 0.05; Table 1); the svPPA and lvPPA groups were on average significantly younger than the healthy control and nfvPPA groups (*p* < 0.05). Patient groups did not differ for mean symptom duration and showed profiles of neuropsychological impairment in keeping with the respective syndromic diagnoses (Table 1). There were no significant differences in peripheral hearing function between participant groups (Table 1).

### 3.2 Post-scan behavioral data

Performance data for the participant groups on the post-scan behavioral tests are presented in Table 1. Patient groups generally performed significantly worse than the healthy control group on these tasks (*p* < 0.05); for the temporal processing task, performance of the svPPA group did not differ significantly from healthy controls (*p* = 0.12).

### 3.3 Functional MRI data

**Fig. 2:**
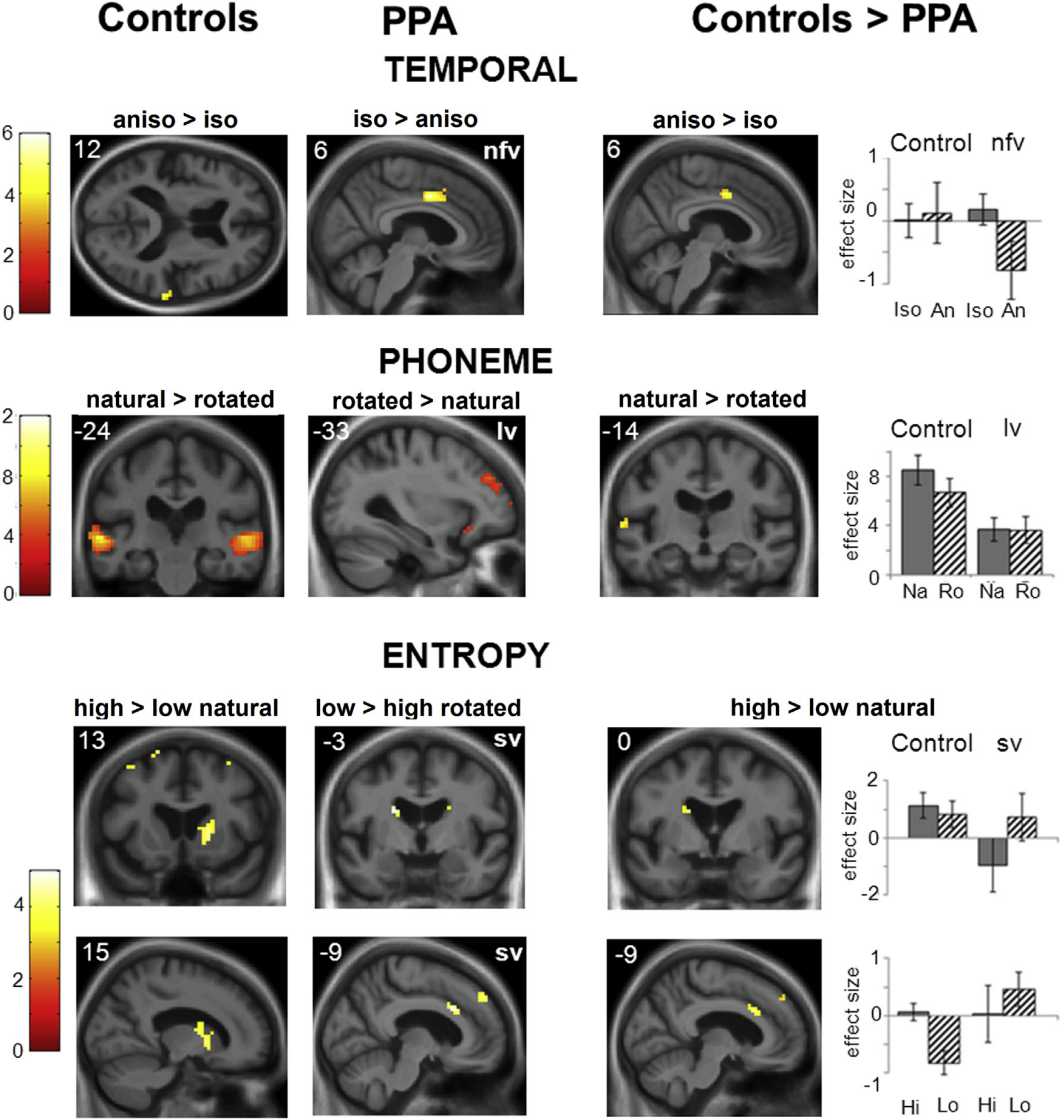
Statistical parametric maps showing fMRI associations of speech signal processing across participant groups. Significant regional brain activations for contrast of interest are shown within healthy control and particular patient groups (left and middle image panels; T scores for relevant contrasts coded in color bars) and between groups (significantly greater activation in healthy controls than the corresponding patient group; right image panels); additional maps showing all significant contrasts are presented in Fig. S3 on-line. Contrasts are coded as follows (see text for details): **temporal**, anisochronous > isochronous conditions (within-controls; controls > nfvPPA), isochronous > anisochronous conditions (within-nfvPPA); **phoneme**, natural > spectrally rotated speech conditions (within-controls; controls > lvPPA), spectrally rotated > natural speech conditions (within-lvPPA); **entropy**, high > low sequence entropy conditions (for natural speech conditions, within-controls; controls > svPPA), low > high sequence entropy conditions (for spectrally rotated speech conditions, within-svPPA). Maps are rendered on representative sections of the study-specific group mean T1-weighted structural MR image in MNI space; the plane of each section is indicated using MNI coordinates and the left cerebral hemisphere is displayed on the left in axial and coronal sections. Maps have been thresholded at *p* < 0.001 uncorrected over the whole brain for display purposes; all activations shown were significant at *p* < 0.05 after family-wise error correction for multiple comparisons (see Table 2). Plots of condition effect size (mean beta parameter estimate ± standard error) are shown (right) for the group comparisons, based on data for peak voxels from the between-group contrasts (see Table 2) in anterior cingulate (temporal contrast), posterior superior temporal gyrus (phoneme contrast), caudate nucleus (entropy contrast, top), and anterior cingulate (entropy contrast, bottom). Abbreviations: An/aniso, anisochronous; Hi, high entropy; Iso, isochronous; Lo, low entropy; lvPPA, logopenic variant primary progressive aphasia; Na/natural, natural speech; nfvPPA, nonfluent variant primary progressive aphasia; Ro/rotated, rotated speech; svPPA, semantic variant primary progressive aphasia. (For interpretation of the references to color in this figure legend, the reader is referred to the Web version of this article.)

Significant neuroanatomical findings from the fMRI analysis are summarized in Table 2; Fig. 2 shows statistical parametric maps and beta parameter estimates for key contrasts and conditions. Additional contrasts are presented in Fig. S3 in Supplementary Material on-line.

**Table 2:** Summary of fMRI associations of speech signal processing across participant groups

Group	Domain	Contrast	Region	Side	Cluster (voxels)	Peak (mm)			t-score	*p*-value
						x	y	z		
Within groups										
Healthy controls	Auditory stimulation	All sound > silence	HG/STG	R	1352	54	−12	0	**14.80**	**<0.001**
			HG/PT	L	1424	−42	−24	6	**14.54**	**<0.001**
			Inferior frontal gyrus	R	45	54	27	18	**4.73**	**0.049**
			Inferior frontal gyrus	L	102	−45	30	12	**4.70**	**0.001**
		Silence > all sound	Precuneus	R	58	21	−63	27	**5.59**	**0.018**
	Temporal regularity	Anisochronous > isochronous	Post STG	R	7	69	−30	9	4.25	0.049
	Phonemic structure	Natural > rotated speech	Post STG/STS	L	739	−60	−12	−3	**10.38**	**<0.001**
			Post STS/Mid STG	R	593	54	−30	3	**8.01**	**<0.001**
			Primary motor	L	69	−51	−6	48	**7.97**	**0.006**
			Primary motor	R	44	45	6	51	**5.80**	**0.045**
	Sequence information	High > low entropy	Caudate^a^	R	54	18	12	3	**4.35**	**0.015**
nfvPPA	Auditory stimulation	All sound > silence	HG/PT	L	938	−60	−18	3	**11.2**	**<0.001**
			HG/PT/post STG/STS	R	936	63	−18	9	**10.4**	**<0.001**
		Silence > all sound	TPO	R	50	42	−60	9	**4.35**	**0.033**
	Temporal regularity	Isochronous > anisochronous	ACC/SMA	R	56	6	3	42	**5.43**	**0.018**
	Phonemic structure	Natural > rotated speech	Post STS/mid STG	L	275	−54	3	−12	**6.26**	**<0.001**
			Post/mid STS	R	257	69	−18	−6	**5.53**	**<0.001**
			Inferior frontal gyrus	L	108	−57	18	12	**4.95**	**<0.001**
			Primary motor	R	52	51	0	48	**4.93**	**0.023**
svPPA	Auditory stimulation	All sound > silence	HG/PT	L	877	−45	−36	12	**11.08**	**<0.001**
			HG/PT/post STG/STS	R	867	63	−30	3	**7.25**	**<0.001**
		Silence > all sound	Post inferior temporal sulcus^c^	R	62	54	−18	−21	**4.40**	**0.013**
	Phonemic structure	Natural > rotated speech	Primary motor	L	48	−51	3	48	**6.53**	**0.032**
			Post STS	R	132	57	−30	3	**5.82**	**<0.001**
			Post STS/mid STS/STG	L	104	−63	−30	−3	**5.68**	**0.001**
			SMA	R	49	6	12	63	**5.20**	**0.030**
			Primary motor	R	67	48	0	45	**4.97**	**0.007**
	Sequence information	High > low entropy	OFC/IFG^b^	R	83	39	57	−15	**4.33**	**0.003**
		Low > high entropy	DLPFC^b^	R	64	18	39	39	**4.81**	**0.012**
			ACC^b^	L	13	−9	21	30	4.41	0.002
			Caudate^b^	L	11	−21	−3	21	4.85	0.009
lvPPA	Auditory stimulation	All sound > silence	HG	L	296	−39	−27	6	**7.95**	**<0.001**
			HG/PT/post STG/STS^c^	R	641	63	−24	0	**6.90**	**<0.001**
	Phonemic structure	Rotated > natural speech	DLPFC^c^	L	76	−33	42	30	**4.90**	**0.004**
Between groups										
Controls > nfvPPA	Auditory stimulation	All sound > silence	Medial HG	R	48	39	−21	12	**5.59**	**0.038**
	Temporal regularity	Anisochronous > isochronous	ACC	R	16	6	3	42	4.65	0.014
Controls > svPPA	Sequence information	High > low entropy	Caudate^b^	L	12	−21	−3	21	4.32	0.006
			ACC^b^	L	12	−9	21	30	5.08	0.004
Controls > lvPPA	Phonemic structure	Natural > rotated speech	Post STG/STS^c^	L	12	−60	−24	0	4.12	0.025

**Fig. S2:**
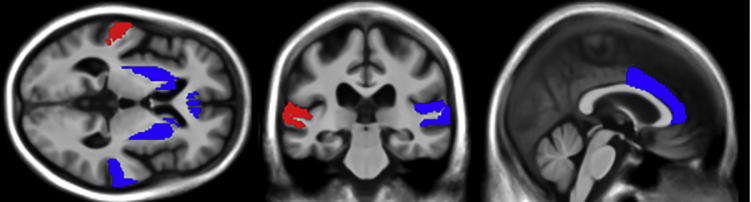
Anatomical small regions used in fMRI analysis. The figure shows representative sections of anatomical regions used for multiple voxel-wise comparisons corrections in region-of-interest analyses based on prior anatomical hypotheses (see text). Bi-hemispheric regions of interest are rendered on sections of the average normalized brain template for the combined patient cohort. The left cerebral hemisphere is shown on the left on the coronal section and above in the axial section. For the contrast assessing phonemic processing, the anatomical region for small volume correction comprised left posterior superior temporal gyrus/sulcus and planum temporale (red areas). For the contrasts assessing temporal processing and sequence information (entropy) processing, the anatomical region of interest comprised this left superior temporal lobe region plus additional regions in right superior temporal lobe, striatum (caudate and putamen), and anterior cingulate cortex (red plus blue areas).

Regional cerebral activations for contrasts of interest in each participant group and between control and patient groups are summarized (see text for details of contrasts). Local maxima significant at *p* < 0.05_FWE_ cluster-level, corrected for multiple voxel-wise comparisons over the whole brain are in bold; other maxima are significant at *p* < 0.05_FWE_ peak-level corrected for multiple comparisons over prespecified anatomical regions of interest (see text and Fig. S2) and coordinates of local maxima are in MNI standard space.

Key: ACC, anterior cingulate cortex; DLPFC, dorsolateral prefrontal cortex; HG, Heschl's gyrus; L, left; lvPPA, patient group with logopenic variant primary progressive aphasia; nfvPPA, patient group with nonfluent variant primary progressive aphasia; OFC, orbitofrontal cortex; Post, posterior; PT, planum temporale; R, right; SMA, supplementary motor area; STG/S, superior temporal gyrus/sulcus; svPPA, patient group with semantic variant primary progressive aphasia; TPO, temporo-parieto-occipital junction.

a Indicates that signal was driven by natural speech condition, or ^b^By spectrally rotated speech condition.

c Indicates region also the site of a local maximum in the VBM analysis of gray matter atrophy (see Table S1).

#### 3.3.1 Auditory stimulation

**Fig. S3:**
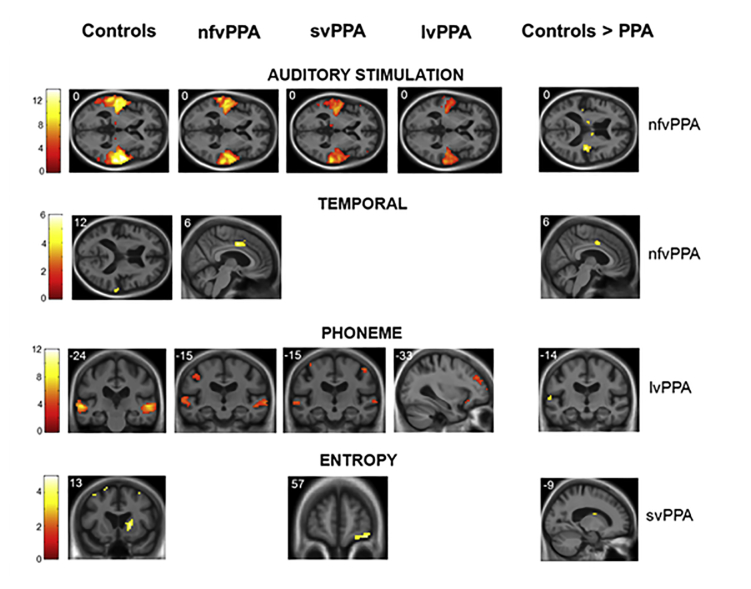
Additional statistical parametric maps representing all fMRI associations of speech signal processing across participant groups. Significant regional brain activations for contrast of interest are shown within healthy control and patient groups (left and middle panels; T scores for relevant contrasts coded in color bars) and between groups (significantly greater activation in healthy controls than the corresponding patient group, indicated beside the panel; right-most panels). Contrasts are coded as follows (see text for details): **Auditory stimulation**, all sound > silence conditions; **temporal**, anisochronous > isochronous conditions (within-controls; controls > nfvPPA), isochronous > anisochronous conditions (within-nfvPPA); **phoneme**, natural > spectrally rotated speech conditions (within-controls, within-nfvPPA, within-svPPA; controls > lvPPA), spectrally rotated > natural speech conditions (within-lvPPA); **entropy**, high > low sequence entropy conditions. Maps are rendered on representative sections of the study-specific group mean T1-weighted structural MR image in MNI space; the plane of each section is indicated using MNI coordinates and the left cerebral hemisphere is displayed on the left in axial and coronal sections. Maps have been thresholded at *p* < 0.001 uncorrected over the whole brain for display purposes; all activations shown were significant at *p* < 0.05 after family-wise error correction for multiple comparisons (see Table 2). Abbreviations: lvPPA, logopenic variant primary progressive aphasia; nfvPPA, nonfluent variant primary progressive aphasia; svPPA, semantic variant primary progressive aphasia.

Auditory stimulation (all sound conditions versus silence) produced extensive bilateral activation of Heschl's gyrus and superior temporal gyrus in all participant groups (all *p* < 0.05_FWE_ over the whole brain; Fig. S3). Certain participant groups showed a significantly greater effect of silence than auditory stimulation in posterior temporo-parietal cortices: the healthy control group showed this effect in precuneus, the nfvPPA group in right temporo-parieto-occipital junction, and the svPPA group in posterior inferior temporal sulcus (all *p* < 0.05_FWE_ over the whole brain). Auditory stimulation produced significantly greater activation of medial Heschl's gyrus in the healthy control group than the nfvPPA group but no other significant group differences emerged at the prescribed threshold (*p* < 0.05_FWE_ over the whole brain).

#### 3.3.2 Temporal isochrony

Processing of temporal irregularity in speech signals (anisochronous vs. isochronous conditions) was associated in the healthy control group with significant activation of right posterior superior temporal gyrus (*p* < 0.05_FWE_ within the pre-specified anatomical region of interest); whereas temporal regularity (isochronous versus anisochronous conditions) was associated in the nfvPPA group with significant activation of right anterior cingulate and supplementary motor cortices (*p* < 0.05_FWE_ over the whole brain; Fig. 2). The effect of temporal irregularity was significantly greater for the healthy control group than the nfvPPA group in anterior cingulate cortex (*p* < 0.05_FWE_ within the prespecified anatomical region of interest; Fig. 2). Plotting parameter estimates for the temporal regularity contrast (Fig. 2) revealed a relative deactivation to anisochronous syllable sequences in the nfvPPA group that was not present in the healthy control group. No other significant group correlates of temporal processing were identified.

#### 3.3.3 Phonemic spectral structure

The presence of phonemic structure (natural vs. spectrally rotated phonemes) was associated with significant bilateral activation of lateral posterior to mid superior temporal gyrus and sulcus and more dorsal motor areas in the healthy control group, the nfvPPA group, and the svPPA group (all *p* < 0.05_FWE_ over the whole brain; Fig. 2). Conversely, the lvPPA group showed no activation in response to phonemic structure at the prescribed threshold but rather significant activation of left dorsolateral prefrontal cortex in response to spectrally rotated speech (*p* < 0.05_FWE_ over the whole brain). The effect of phonemic structure in left posterior superior temporal cortex was significantly greater for the healthy control group than the lvPPA group (*p* < 0.05_FWE_ within the prespecified anatomical region of interest), driven by increased activation in response to natural speech in healthy controls that was not present in patients with lvPPA (Fig. 2).

#### 3.3.4 Signal information content (entropy)

Increasing signal information content (high vs. low sequence entropy) in natural speech sequences was associated with significant activation of right caudate nucleus in the healthy control group (*p* < 0.05_FWE_ over the whole brain; Fig. 2); none of the patient groups showed a significant effect for this contrast, whereas healthy controls showed no significant effect for spectrally rotated speech conditions at the prescribed threshold. However, for spectrally rotated speech conditions, the svPPA group showed significant activation of right orbitofrontal cortex and inferior frontal gyrus in response to increasing signal information content (*p* < 0.05_FWE_ over the whole brain, Fig. S3) and significant activation of right dorsolateral prefrontal cortex, left anterior cingulate, and left caudate in response to reduced signal information content (low vs. high sequence entropy; *p* < 0.05_FWE_ within the prespecified anatomical region of interest, Fig. 2). The effect of increasing signal information was significantly greater in the healthy control group than the svPPA group (*p* < 0.05_FWE_ within the prespecified anatomical region of interest), driven by relative deactivation of left caudate in the high-entropy condition and the activation of anterior cingulate cortex in the low entropy condition in the patients with svPPA (Fig. 2).

### 3.4 Correlations of functional neuroanatomical with post-scan behavioral data

Performance on the post-scan test of phoneme processing was significantly positively correlated with peak activation of left superior temporal gyrus across the lvPPA and healthy control groups (t(19) = 4.08, *p* = 0.001, R^2^ = 0.47), though this was not significant within the lvPPA group (t(4) = 0.68, *p* = 0.53, R^2^ = 0.10). Performance on the post-scan test of entropy processing was significantly inversely correlated with peak activation of left caudate (t(21) = 3.38, *p* = 0.003, R^2^ = 0.35) and left anterior cingulate (t(21) = 3.42, *p* = 0.003, R^2^ = 0.35) across the svPPA and healthy control groups, though not significant within the svPPA group (t(6) = 1.62, *p* = 0.16, R^2^ = 0.30 in left caudate and t(6) = 0.94, *p* = 0.38, R^2^ = 0.13 in left anterior cingulate). There were no significant functional neuroanatomical correlations with performance on the post-scan temporal processing test.

### 3.5 Comparison of functional with structural neuroanatomical data

**Fig. S4:**
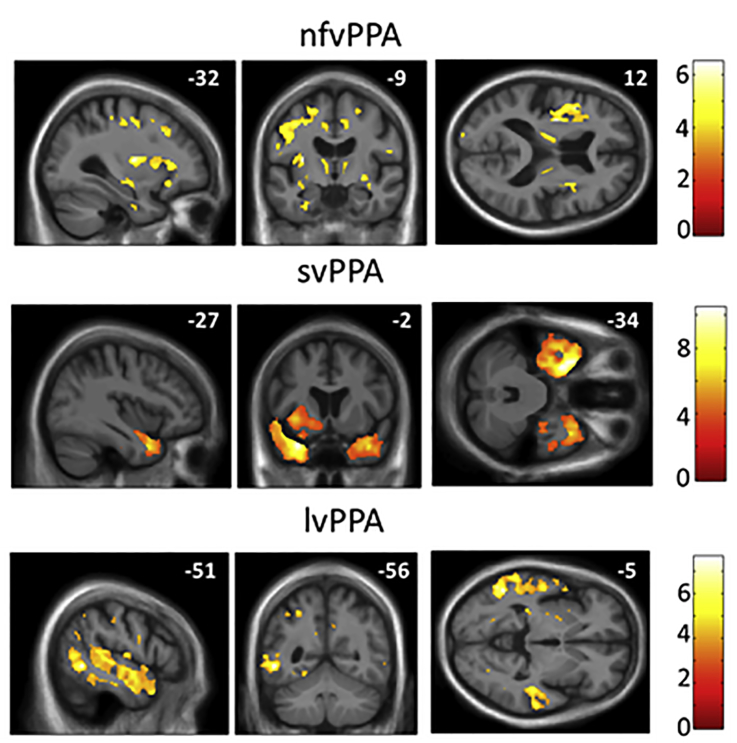
Statistical parametric maps showing disease-related structural neuroanatomical differences between each patient group and the healthy control group, from the voxel-based morphometry analysis. Maps are thresholded at *p* < 0.001 uncorrected over the whole brain and displayed on representative sections of the group mean T1-weighted template image in MNI space; the plane of each section is indicated using MNI coordinates and the left cerebral hemisphere is displayed on the left in axial and coronal sections (color bars code T scores for the relevant atrophy map). Abbreviations: lvPPA, logopenic variant primary progressive aphasia; nfvPPA, nonfluent variant primary progressive aphasia; svPPA, semantic variant primary progressive aphasia.

Each of the patient groups showed the anticipated profile of disease-related gray-matter atrophy; statistical parametric maps are presented in Fig. S4 and structural neuroanatomical correlates are summarized in Supplementary Table S1. Comparing the distribution of local maxima for gray-matter atrophy and functional activation (Table 2), a common regional locus was identified in posterior superior temporal cortex for the comparison of the lvPPA and healthy control groups; no other coincident gray-matter regions were identified for the key comparisons between patients and healthy controls. Within patient groups, additional common regional correlates of atrophy and functional activation were identified for the auditory stimulation contrast in right posterior inferior temporal cortex within the svPPA group and in right auditory cortex within the lvPPA group; across syndromic groups, peak functional, and structural regional gray matter correlates were largely noncontiguous (Table 2).

## 4 Discussion

We have shown that canonical PPA syndromes are associated with distinctive functional neuroanatomical profiles of abnormal speech signal decoding relative to healthy older individuals. Compared directly with the healthy control group, the nfvPPA group showed reduced activation of medial Heschl's gyrus in response to auditory stimulation (across all sound conditions) and reduced activation of anterior cingulate cortex in response to temporal irregularity in speech sequences. The svPPA group showed reduced activation of caudate and anterior cingulate in response to increased signal information content (entropy) in spectrally rotated speech. The lvPPA group showed reduced activation of posterior superior temporal cortex in response to phonemic spectral structure. These syndromic signatures are in accord with prior predictions concerning the informational components of speech signals that are most likely to be vulnerable in each PPA syndrome (Golden et al., 2015, Grube et al., 2016, Hailstone et al., 2012, Hardy et al., 2015, Henry et al., 2016, Hsieh et al., 2011, Lambon Ralph et al., 2010, Rohrer et al., 2010). Performance on post-scan behavioral testing correlated with regional neural activation for the processing of phonemic structure and signal information content for the relevant syndromic (lvPPA and svPPA) groups relative to healthy controls: functional neuroanatomical profiles may therefore underpin behavioral speech processing deficits in these syndromes, though the lack of correlation within the respective patient groups suggests that additional factors may drive individual performance variation. In general, the distributions of peak functional changes (in particular, those differentiating patients from healthy controls) were not contiguous with maps of peak regional disease-related atrophy, suggesting that cerebral volume loss alone did not drive the functional neuroanatomical profiles observed in the patient groups.

### 4.1 Processing of spectrotemporal structure in speech signals

In response to overall auditory stimulation, the healthy older group and each of the patient groups showed the anticipated extensive activation of primary and association auditory cortices (Binder et al., 2000, Dehaene-Lambertz et al., 2005, Dhamala et al., 2003, Goll et al., 2012, Greicius et al., 2003, Griffiths and Warren, 2002, Liebenthal et al., 2005, Scott et al., 2000). Only the nfvPPA group showed a profile of activation to sound that differed significantly from healthy controls: this is in keeping with emerging evidence for deficits of early auditory perceptual processing in nfvPPA that may distinguish it from other PPA syndromes (Goll et al., 2010, Goll et al., 2011, Grube et al., 2016, Maruta et al., 2014).

More selective alterations emerged for the processing of temporal irregularity in syllable sequences. The healthy control group exhibited an activation profile in line with previous work in the healthy brain showing that auditory rhythmic variation engages posterior superior temporal cortices (Griffiths et al., 1999, Rauschecker and Scott, 2009). None of the patient groups showed enhanced activation in response to syllable anisochrony; conversely, the nfvPPA group showed a distinctively reduced response to anisochronous relative to isochronous syllable sequences in anterior cingulate and supplementary motor cortices. In the healthy brain, this medial prefrontal cortical region is engaged in tracking and integration of temporal patterns embodied in speech syntax and prosody (Hertrich et al., 2016). In nfvPPA, a similar region has been implicated in the pathophysiology of both speech production and rhythm processing deficits, participating in a neural network including inferior frontal gyrus (Ballard et al., 2014, Catani et al., 2013, Schaeverbeke et al., 2016). In light of emerging formulations linking temporal perceptual to output processes both in the healthy brain and in nfvPPA (Grube et al., 2016, Schaeverbeke et al., 2016, Warren et al., 2005), the present finding in this syndromic group may signify a dysfunctional mechanism mediating the sensorimotor transformation of speech signals. It is noteworthy that this activation profile was not correlated with out-of-scanner perceptual assessment of speech stimuli and was moreover right-lateralized, perhaps indicating motor recoding of syllable timings or recruitment of a generic mechanism for decoding signal regularities (Nastase et al., 2014).

For the detection of phonemic spectral structure, the healthy control group showed preferential activation of lateral posterior and mid superior temporal cortex for natural versus spectrally rotated speech. This region of association auditory cortex has been identified as a seat of phoneme processing in the healthy brain (Hickok and Poeppel, 2007, Leaver and Rauschecker, 2010, Liberman and Mattingly, 1989, Obleser et al., 2010, Rauschecker and Scott, 2009; Scott et al., 2000, Scott et al., 2009, Zhang et al., 2016). Neural mechanisms instantiated in this region are likely to be essential (as in the present experiment) for the disambiguation of speech from complex nonspeech sounds at the level of auditory object (phoneme) representation. These mechanisms are bi-hemispherically distributed and left hemisphere specialization may be in part directed by connectivity changes under linguistic tasks (Leaver and Rauschecker, 2010, Markiewicz and Bohland, 2016, Obleser et al., 2010, Zhang et al., 2016). This interpretation also accords with the differential activation profiles shown by the present patient groups on the relevant phonemic contrast: compared with healthy controls, the nfvPPA and svPPA groups showed relatively normal activation profiles, whereas the lvPPA group exhibited a significantly attenuated response to natural phonemes in the key superior temporal region, in line with the clinical deficits of phonological processing (Gorno-Tempini et al., 2008, Grube et al., 2016, Hailstone et al., 2012, Hardy et al., 2015, Henry et al., 2016, Rohrer et al., 2010) and related deficits of paralinguistic analysis (Rohrer et al., 2012) previously documented in lvPPA. Although we did not assess working memory directly in this experiment, posterior superior temporal cortex has been shown to play an integral role in auditory working memory for phonemes as well as other auditory objects (Kumar et al., 2016, Markiewicz and Bohland, 2016), suggesting that the profile identified here is relevant to the phonological working memory impairment that is a defining feature of lvPPA (Gorno-Tempini et al., 2008, Gorno-Tempini et al., 2011). Clinically, phonological deficits are a feature of nfvPPA as well as lvPPA (Hailstone et al., 2012, Hardy et al., 2015, Henry et al., 2016, Rohrer et al., 2010): the present findings suggest that these deficits may have different mechanisms in the 2 syndromes, since the relevant experimental contrast isolated a stage of phonological object representation that is likely to be core to lvPPA rather than nfvPPA (Rohrer et al., 2010). This posterior-superior temporal cortical region was a focus of peak atrophy in the VBM analysis of the lvPPA group. While care is needed interpreting functional changes in the setting of regional atrophy, it is noteworthy that differential activation in this patient group relative to healthy controls was driven by an attenuated response to natural (but not spectrally rotated) speech. This implies that the group-wise activation difference was at least partly attributable to a functionally selective mechanism, rather than simply a nonspecific consequence of gray-matter loss.

Processing of natural speech was additionally associated in the healthy control, nfvPPA, and svPPA groups with prefrontal and motor activation, consistent with obligatory engagement of the dorsal language processing network previously implicated in phonological processing (Warren et al., 2005); in contrast, the lvPPA group showed a paradoxically enhanced response to spectrally rotated speech in dorsal prefrontal cortex. Reduced capacity to integrate spectrotemporal information into auditory object-level representations could potentially underpin both phonological and nonverbal auditory deficits in lvPPA (Golden et al., 2016, Goll et al., 2011, Rohrer et al., 2012) and may be relatively specific for this syndrome, perhaps aligning lvPPA with the auditory apperceptive deficit described in typical Alzheimer's disease (Golden et al., 2016, Goll et al., 2011).

### 4.2 Processing of fundamental information content in speech signals

The processing of signal information content (entropy) in syllable sequences further stratified the healthy control and patient groups. In the healthy control group, increased entropy in natural speech signals engaged right caudate nucleus: this corroborates previous work in the healthy brain implicating striatum in the obligatory tracking of sequence entropy (Nastase et al., 2015, Overath et al., 2007) and more broadly in predictive and probabilistic encoding of speech and other stimuli (Geiser et al., 2012, Grahn and Rowe, 2013, Haruno and Kawato, 2006, Kotz et al., 2009). The nfvPPA and lvPPA groups showed no significant activation in response to the entropy manipulation, whereas this null result should be interpreted with caution (given that no significant differences were identified in these syndromic groups with respect to the healthy control group), sensitivity to the long-range structure of speech signals might plausibly be reduced in PPA syndromes characterized by impaired integration of auditory features unfolding over time (Golden et al., 2016, Hailstone et al., 2012, Rohrer et al., 2012).

A clearer profile of abnormal entropy processing was evident, as predicted, in the svPPA group here. The brain regions engaged in decoding signal entropy in this patient group constitute a distributed fronto-cingulo-striatal network for processing signal statistics in the healthy brain (Fan, 2014). The svPPA group showed responses preferentially for the high entropy condition in inferior frontal cortex, previously shown to be sensitive to increasing uncertainty in speech signals (Nastase et al., 2014); and preferentially for the low entropy condition in caudate, dorsolateral prefontal cortex, and anterior cingulate, regions that have shown more complex responses to varying signal predictability in previous work (Nastase et al., 2014, Nastase et al., 2015). In healthy individuals, anterior cingulate cortex has been implicated in predictive coding and analysis of deviance in auditory and other stimuli (Ide et al., 2013, Kiehl et al., 2000, Lee et al., 2011, Magno, 2006). However, the response profile of the svPPA group differed qualitatively and quantitatively from the healthy control group: qualitatively, patients with svPPA showed sensitivity to entropy variation in spectrally rotated but not natural speech; and quantitatively, these patients showed lower overall sensitivity to increasing signal entropy due to a bidirectional profile of altered activation within the cingulo-striatal network (Fig. 2). Damage involving this network (beyond the signature involvement of anterior temporal cortex) has been demonstrated in svPPA (Rohrer et al., 2009). Moreover, as anterior cingulate cortex mediates widespread shifts in connectivity between distributed brain regions (Crottaz-Herbette and Menon, 2006, Nastase et al., 2014, Nastase et al., 2015), our findings leave open the possibility that altered connectivity to temporal lobe and other structures may have contributed to the behavioral correlate (performance on the out-of-scanner entropy processing task) here.

In information processing terms, these findings illuminate an essential operation in sensory signal analysis that is critically vulnerable in svPPA: the computation of coherent object concepts (Lambon Ralph et al., 2010). Although we only explored auditory processing in the present study, this operation is relevant to object processing in any sensory modality and goes beyond the moment-to-moment perceptual coding of sensory data and detection of ‘patterns’ to extract global statistical regularities in the signal. Signal information of this statistical kind might be used to determine membership of a sensory object category and to identify and predict correspondences between signals in different sensory modalities: a basic requirement for semantic concept formation and evaluation. Indeed, current models of semantic cognition emphasize the graded and predictive nature of object concepts and the problem of integrating object information cross-modally into coherent multi-modal concepts (Lambon Ralph et al., 2017). Based on the present data in svPPA, we propose that signal entropy accesses a generic neural algorithm that computes and predicts sensory object attributes for further semantic analysis. Interpreted in these terms, the lack of a differential effect of entropy conditions in the nfvPPA and lvPPA groups here would be consistent with a more fundamental impairment of pitch pattern analysis in these syndromes, whereas the differential entropy effect indexed in the svPPA group would reflect a disproportionate deficit in computing object-level statistics in svPPA (Golden et al., 2015, Golden et al., 2016, Hsieh et al., 2011, Lambon Ralph et al., 2010, Rohrer et al., 2012).

### 4.3 Neurobiological and clinical implications

From a neurobiological perspective, this study has uncovered defective brain mechanisms for decoding auditory speech signal attributes (temporal structure, spectral structure, and information content) that are likely to underpin particular PPA syndromes (nfvPPA, lvPPA, and svPPA, respectively). Considered collectively, the findings suggest a common pathophysiological theme in these syndromes. Efficient decoding mechanisms in the healthy brain use fewer computational (physiological) resources when less information is present in the sensory signal (Overath et al., 2007): it is noteworthy that each of the PPA syndromes here (in the key contrast signifying that syndrome) reversed this normal pattern. This was most clearly the case for svPPA (in which “low information” [entropy] stimulus conditions evoked more activity in relevant brain regions), but analogous inefficiency may also account for the greater response to isochronous than anisochronous stimuli in nfvPPA and the loss of the processing advantage for natural speech in lvPPA. Reduced computational efficiency of cortical information processing may be pathophysiologically relevant to many neurodegenerative proteinopathies (Warren et al., 2013): increased metabolic demands related to reduced efficiency may be a mechanism of neural network vulnerability in these diseases. Bayesian accounts of the brain as an engine for minimizing prediction errors about the world at large and disease effects on this predictive coding are gaining wide currency (Adams et al., 2013, Barascud et al., 2016, O'Reilly et al., 2013). In Bayesian terms, loss of computational efficiency in PPA syndromes might plausibly be associated with imprecise coding of speech and other auditory patterns and therefore less reliable detection of unexpected, deviant, or irregular auditory events. It is noteworthy that the auditory cortical and prefrontal areas identified as differentially active in our patient groups participate in predictive sensory coding in the healthy brain (Barascud et al., 2016, O'Reilly et al., 2013).

From a clinical perspective, the identification of pathophysiological mechanisms using fMRI has several implications. Functional MRI can identify aberrant increases as well as reductions in cerebral activity (see Fig. 2) and functional alterations remote from the foci of atrophy (see Table 2): in the context of a clinical trial, incorporation of an activation fMRI limb might allow detection of dynamic therapeutic effects on working brain function that are not captured by conventional structural or even resting-state fMRI techniques. More broadly, fMRI provides a neuroanatomical grounding for behavioral measures (such as phonemic processing in lvPPA and entropy processing in svPPA) that correlate with brain network changes in particular syndromes: such surrogate behavioral measures could yield new, translatable biomarkers that both capture core pathophysiology and do not depend on conventional neurolinguistic tests.

### 4.4 Conclusions and future directions

This study has several limitations that suggest opportunities for future work. The PPA syndromes are clinically and pathologically heterogeneous and evolve dynamically over time, typically with convergence between syndromes (Josephs and Duffy, 2008, Josephs et al., 2013, Rohrer et al., 2010, Seelaar et al., 2011); larger cohorts studied prospectively and ultimately, with molecular and/or histopathological correlation might enable further pathophysiological stratification of syndromes and assessment of the value of fMRI signatures in tracking and forecasting disease evolution. Combining neuroanatomical modalities might yield further perspectives on these issues: it is likely, for example, that the temporal signature of signal processing will be sensitive to the effects of PPA pathologies, and this could be captured using a technique such as magnetoencephalography (Wibral et al., 2011). The present fMRI paradigm was based on passive listening: in future studies, it will be important to determine the extent to which the functional neuroanatomical profiles demonstrated here are modulated in the context of an output task. This speaks to the relevance of such profiles to the symptoms and capacities that patients exhibit in their everyday lives: further work is required to determine how functional neuroanatomy relates to neurolinguistic deficits and to measures of daily-life disease burden. At the same time, it would likely be informative to sample a wider range of speech signal characteristics: this study employed a limited range of phonemic carriers and future work could explore the effect of a more representative set and examine the interaction of phoneme identity with other experimental parameters.

Acknowledging the caveats above, this work identifies candidate signal processing operations that may be core to particular PPA syndromes and suggests a generic pathophysiological mechanism of reduced neural computational efficiency and precision in these proteinopathies, extending beyond the disintegrating language network. Speech is, essentially, a species of complex sound. In light of the nosological difficulties that surround PPA and mounting neuropsychological and functional neuroanatomical evidence for nonverbal auditory impairment in these syndromes (Golden et al., 2015, Golden et al., 2016, Goll et al., 2010, Goll et al., 2011, Goll et al., 2012, Grube et al., 2016, Hailstone et al., 2011, Hailstone et al., 2012, Hardy et al., 2016, Rohrer et al., 2012), it may be timely to reevaluate the ‘language-led’ dementias as more fundamental disorders of signal decoding. This in turn could have important implications for the development of new biomarkers, diagnostic formulations, and therapeutic interventions.

## Disclosure statement

The authors have no conflicts of interest to disclose.

## Figures and Tables

**Fig. S1:**
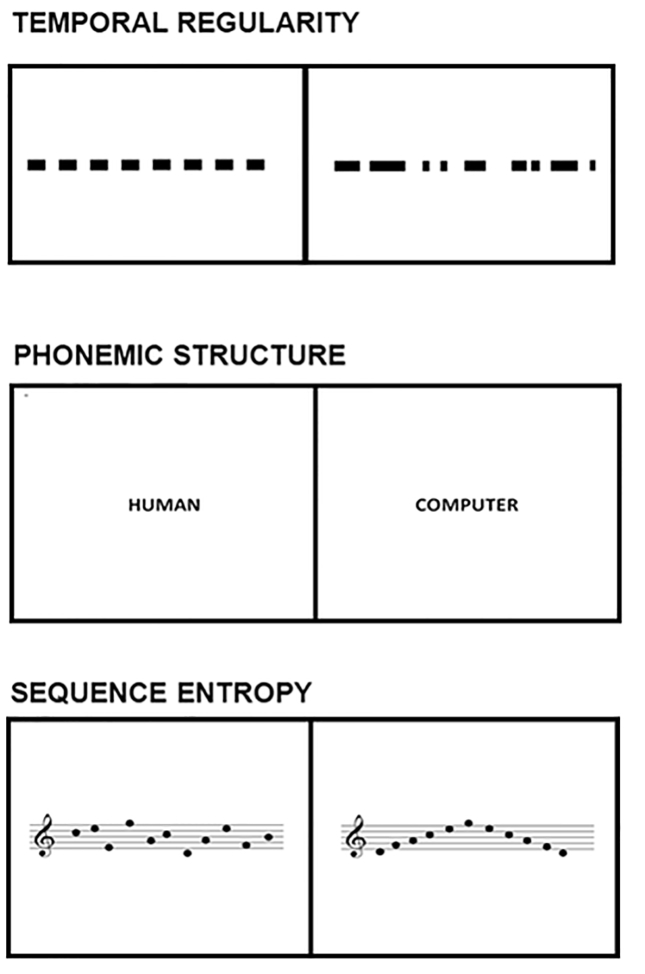
Visual aids used in post-scan behavioral testing. Pictorial cue cards were used to assist understanding and responding on the post-scan behavioral tasks. For the test assessing temporal processing (top panels), on each trial participants were asked to decide whether the sounds they heard came regularly or irregularly. For the test assessing processing of phonemic structure (middle panels), participants were asked to decide whether the sounds were made by a human or by a computer. For the test assessing processing of sequence entropy, participants were asked to decide whether the sounds were arranged randomly or following a pattern. On each trial, participants were able to respond verbally or by pointing to the appropriate cue card.

## Appendix A Supplementary data

Hardy Supplementary Audio Natural-Isochronous-High Entropy

Hardy Supplementary Audio Rotated-Anisochronous-Low Entropy
