# Supplementary material for: Functional neuroanatomy of speech signal decoding in primary progressive aphasias
Source: Neurobiol Aging. 2017 Aug;56:190–201. doi: 10.1016/j.neurobiolaging.2017.04.026 (PMC5476347; doi:10.1016/j.neurobiolaging.2017.04.026)
Supplement: Table S1 [file mmc3.docx]

**SUPPLEMENTARY MATERIAL**

**Functional neuroanatomy of speech signal decoding in primary progressive aphasias,**

**by CJD Hardy et al**

**Procedure for assessing peripheral hearing**

Pure tone audiometry was performed using an Otovation Roto audiometer ([www.otovation.com](http://www.otovation.com)) in a quiet room. Five frequency levels were tested (500, 1000, 2000, 4000, 6000 Hz). At each frequency, the participant was played three tones, starting at 20dB. If the participant indicated correctly that they had heard at least two of the three tones, this was recorded as the threshold for that frequency; if not, the level was increased in increments of 5dB up to 70dB. Hearing was assessed in both ears for each participant.

**Table S1.** Regions of grey matter atrophy in the PPA groups.

| **Region** | **Side** | **Cluster**  (voxels) | **Peak** (mm) | | | ***t*-value** |
| --- | --- | --- | --- | --- | --- | --- |
|  |  |  | **x** | **y** | **z** |  |
| **nfvPPA** |  |  |  |  |  |  |
| Lingual gyrus | L | 383 | -14 | -56 | 2 | 6.46 |
| Putamen/ insula | L | 2121 | -32 | -9 | 12 | 6.31 |
| Superior frontal gyrus | L | 2910 | -18 | -6 | 58 | 6.01 |
| Middle occipital gyrus | R | 128 | 33 | -76 | 8 | 5.73 |
| Supplementary motor cortex | L | 1214 | -10 | 6 | 58 | 5.59 |
| Lingual gyrus | R | 1045 | 21 | -46 | -4 | 5.58 |
| Thalamus | L | 666 | -16 | -16 | 10 | 5.23 |
| Posterior orbital gyrus | L | 107 | -27 | 26 | -14 | 5.10 |
| Middle temporal gyrus | L | 115 | -52 | -54 | -4 | 5.03 |
| Postcentral gyrus | R | 849 | 36 | -26 | 50 | 4.91 |
| Putamen/ insula | R | 174 | 32 | 8 | 12 | 4.90 |
| Paracingulate gyrus | R | 301 | 10 | -3 | 48 | 4.64 |
| Hippocampus | L | 177 | -30 | -14 | -10 | 4.59 |
| Thalamus | R | 163 | 21 | -20 | 10 | 4.40 |
| Fusiform gyrus | L | 109 | 28 | -9 | -34 | 4.32 |
| **svPPA** |  |  |  |  |  |  |
| Temporal pole | L | 22134 | -27 | 2 | -34 | 10.43 |
| Temporal pole/ inferior temporal sulcus* | R | 4231 | 39 | 9 | -33 | 6.76 |
| Posterior middle temporal gyrus | L | 169 | -60 | -57 | 8 | 4.91 |
| Temporo-parietal-occipital junction | L | 166 | -42 | -62 | 6 | 4.86 |
| **lvPPA** |  |  |  |  |  |  |
| Middle/ superior temporal gyrus | L | 9553 | -51 | -56 | -4 | 7.61 |
| Middle/ superior temporal gyrus* | R | 1462 | 62 | -21 | -8 | 7.25 |
| Precuneus | L | 459 | 0 | -68 | 34 | 7.04 |
| Superior parietal lobule | L | 857 | -30 | -45 | 42 | 6.81 |
| Hippocampus | L | 125 | -33 | -26 | -8 | 5.77 |
| Fusiform gyrus | L | 415 | -32 | -32 | -27 | 5.29 |
| Middle occipital gyrus | L | 270 | -33 | -80 | 26 | 5.20 |
| Temporal pole | R | 213 | 40 | 8 | -30 | 5.04 |
| Posterior insula | R | 110 | 34 | -6 | -15 | 4.98 |
| Posterior cingulate | R | 419 | -4 | -28 | 42 | 4.76 |
| Middle frontal gyrus | L | 183 | -24 | 32 | 38 | 4.55 |
| Fusiform gyrus | R | 181 | 50 | -22 | -22 | 4.49 |
| Dorsolateral prefrontal cortex* | L | 129 | -30 | 42 | 27 | 4.39 |

Regions of significant regional grey matter atrophy in each patient group compared with the healthy control group from the voxel-based morphometry analysis are presented (see also Figure S4). Associations shown are significant at threshold p < 0.001 uncorrected for multiple comparisons over the whole brain; all significant clusters > 100 voxels are shown and peak (local maxima) coordinates are in MNI space. *indicates region also the site of an fMRI loci (see Table 2)
